# Antibiotic Consumption Patterns in European Countries May Be Associated with the Incidence of Major Carcinomas

**DOI:** 10.3390/antibiotics9100643

**Published:** 2020-09-25

**Authors:** Gábor Ternák, Károly Berényi, András Sümegi, Ágnes Szenczi, Barbara Fodor, Balázs Németh, István Kiss

**Affiliations:** 1 Institute of Migration Health, Medical School, University of Pécs, Szigeti st. 12., H-7624 Pécs, Hungary; 2 Department of Public Health Medicine, University of Pécs, Szigeti st. 12., H-7624 Pécs, Hungary; karoly.berenyi@aok.pte.hu (K.B.); sumegia@sumegia.com (A.S.); agnes.szenczi@aok.pte.hu (Á.S.); fodor.barbara92@gmail.com (B.F.); balazs.nemeth@aok.pte.hu (B.N.); istvan.kiss@aok.pte.hu (I.K.)

**Keywords:** cancer, cancer incidence, carcinogenesis, cancer types, antibiotic consumption, intestinal microbiome, dysbiosis, correlation, significant

## Abstract

The possible role of the altered intestinal microbiome in the development of malignancies has been raised recently in several publications. Among external factors, antibiotics are considered to be the most important agent capable of producing dysbiosis in the gut flora, either temporally or permanently. The human microbiome has several beneficial effects in terms of maintaining appropriate human health, but its alteration has been implicated in the development of many illnesses. Our basic aim was to explore a possible relationship between the consumption of different antibiotic classes and the incidence of the most common cancer types (male, female) in European countries. A database of the average, yearly antibiotic consumption (1997–2018) has been developed and the consumption figures were compared to the eight, most frequent cancer incidence calculated for 2018 in 30 European countries. Pearson correlation has indicated different degrees of positive (supportive) and negative (inhibitor) significant associations between antibiotic consumption figures and cancer prevalence. It has been observed that certain antibiotic classes with positive correlation probably augment the incidence of certain cancer types, while others, with negative correlation, may show some inhibitory effect. The relatively higher or lower consumption pattern of different classes of antibiotics could be related to certain cancer prevalence figures in different European countries. Our results indicated that countries with relatively high consumption of narrow-spectrum penicillin (J01CE, J01CF) and tetracycline (J01A), like certain Scandinavian countries, showed a higher incidence of female colorectal cancer, female lung cancer, melanoma, breast, prostate and uterus corpus cancer. Countries with relatively higher consumption of broad-spectrum penicillin (J01CA, J01CR) and some broad-spectrum antibiotics (J01D, J01F, J01M), like Greece, Hungary, Slovakia, France, etc. showed a higher incidence rate of male lung cancer and male bladder cancer. The higher incidence rate of different cancer types showed association with the higher consumption of antibiotics with “augmenting” properties and with less consumption of antibiotics with “inhibitory” properties.

## 1. Introduction

The first known cancer cases in humans have been verified in intact mummies from the pharaonic necropolis of Qubbet el-Hawa in Aswan, Egypt (BC 2000–1800), when CT scan was performed on the bodies. Researchers identified breast cancer and multiple myeloma [1].

As of now, 3.9 million new cancer cases and 1.9 million cancer deaths were estimated in Europe in 2018. Cancers of the female breast (523,000 new cases, 13% of all cancer cases), colorectum (500,000, 13%), lung (470,000, 12%) and prostate (450,000, 12%) were the most common cancers on the continent and combined they represented almost half of the overall cancer burden [2].

The transformation from a normal cell into a tumor cell is a multistage process in which growths often invade surrounding tissues and can metastasize to distant sites. Apart from the genetic background, several external agents with carcinogenetic properties, such as chemicals, toxins, irradiations, infections, etc. can contribute to the process. Recently, the possible role of an altered intestinal microbiome has been raised in the process of carcinogenesis by several researchers and the possible effect of certain antibiotics has been mentioned [3,4,5,6,7,8,9]. Tumor-promoting effects of the microbiota in colorectal cancer (CRC) seem to be caused by altered host–microbiota interactions and by dysbiosis, rather than by infections with specific pathogens. Accordingly, germ-free status and treatment with wide-spectrum antibiotics led to a significant reduction of the numbers of tumors in chemical and genetic experimental models of colorectal carcinogenesis. Hence, the strong microbiome–modification capability of antibiotics and their indirect role in the development of malignancies should be considered [10].

It has been reported that increased exposure to antibiotics during 15 years was associated with a significant increase in prostate cancer risk. This association was dose-dependent [11]. For gastrointestinal malignancies, the use of penicillin was associated with an elevated risk of esophageal, gastric and pancreatic cancers [12]. Similarly, a dose-dependent increase in breast cancer risk was observed in association with antibiotic exposure up to 15 years in the past [13]. Accumulating evidence from animal models suggests that specific microbes and microbial dysbiosis can potentiate hepatobiliary–pancreatic tumor development by damaging DNA, activating oncogenic signaling pathways and producing tumor-promoting metabolites [14].

## 2. Working Hypotheses/Concept

Based on the extensive documentation of the possible role of the altered microbiome in the carcinogenesis, it may be concluded that agents, like antibiotics, having strong potency of producing alteration of the microbiome, could trigger certain pathologic processes, leading to the development of different malignancies. Substantial variation of cancer incidence and mortality rates are observed at the national level across EU countries and it is logical to suspect that similar variations of the causative agents, including the alteration of the microbiome, may be the driving force behind this phenomenon. Considering the possible role of microbiome-triggered mechanisms in carcinogenesis, we hypothesize that the consumption of different antibiotics, generating different modifications of the microbiome, can contribute to the process of carcinogenesis. The hypothesis suspects the association of antibiotic consumption patterns and cancer morbidity data (prevalence, incidence) in 30 European countries included in the study.

## 3. Materials and Methods

Incidence data of the most frequent cancer types in males and females (breast, colorectal, lung, melanoma, prostate, uterus corpus, bladder, kidney), in 30 European countries had been statistically compared to average, yearly antibiotic consumption patterns in the same countries (Table 1) [2,15,16,17,18,19].

Antibiotic consumption database for comparison has been extracted from the ECDC yearly reports on antibiotic consumption for the years of 1997 to 2018 (22 years) reported from 30 EU countries included in the study [17]. The amount of antibiotic consumption appeared as defined daily dose (DDD) per 1000 inhabitants per day (DID) in the respective countries. Average yearly antibiotic consumptions were calculated for 1997–2018 (22 years) in DID as the total systemic antibiotic consumption (J01) and at ATC (Anatomic Therapeutic Chemical classification) [14] classification Level 3 for the major classes of antibiotics as J01A (tetracycline), J01C (penicillin), J01D (cephalosporin), J01F (macrolide), J01 M (quinolone) and at ATC Level 4 for the narrow-spectrum, penicillinase-sensitive penicillin (J01CE), penicillinase-resistant narrow-spectrum penicillin (J01CF), broad-spectrum beta-lactamase sensitive penicillin (J01CA) and broad-spectrum combined with beta-lactamase inhibitors (J01CR). The total systemic antibiotic consumption (J01) expressed in DID/countries were considered as 100% of the respective antibiotic consumption in the countries included in the study and the amount of antibiotic consumption of J01A, J01C, J01D, J01F, J01M, has been calculated as relative share of the total amount (J01) and expressed in percentage (%). Similarly, all the subgroups of penicillin (J01CE, J01CF, J01CA and J01CR) at ATC Level 4 has been calculated as relative share of the total consumption (J01) and expressed in percentage. Groups of narrow (J01CE+CF) and broad (J01CA+CR)-spectrum penicillin were formulated and featured as cumulative relative share of the total amount of systemic consumption (Table 2).

Rank order (decreasing) with the highest incidence of different cancer types (first 10 countries) has been compared to the rank order of antibiotic consumption data (decreasing) to observe the similarities between the higher cancer incidence and the higher consumption figures of antibiotics probably facilitating the development of cancers. Invers (increasing) rank order of antibiotics with supposedly inhibitor effect on the development of cancer was similarly compared to the rank order of cancer incidence (Table 3, Table 4, Table 5, Table 6, Table 7 and Table 8).

### Statistics

Pearson’s correlation was applied for calculating correlation and statistical significance. Positive correlation and significance were estimated when the *p* value was ≤0.05 and the r was a positive number. Negative (inverse) significance was estimated when the *p* value was ≤0.05 and the r was a negative number.

## 4. Results

All types of cancers included in the study showed significant or statistically insignificant, positive or negative associations with at least one, ore more classes of antibiotics, including with the total consumption (J01) or with the high consumption rate of broad-spectrum antibiotics (J01 B/N). It is of importance that the male and female cancer patients within the same location (colorectal, lung, melanoma, kidney, bladder) in certain cases, showed differently, sometimes opposite, associations with the same antibiotic groups.

### 4.1. Colorectal Cancer

Male patients showed a statistically insignificant negative correlation with tetracycline consumption (J01A), and no other associations were observed. In female cases, the statistically insignificant, negative association was found with the total consumption of systemic antibiotics (J01) and quinolone (J01M) consumption and significant negative (inhibitor) association with cephalosporin (J01D). Strong, positive, supportive, significance was found with the consumption of narrow-spectrum penicillin (J01CE, J01CF).

### 4.2. Lung Cancer

In males, positive significance was found with broad-spectrum penicillin (J01CA + J01CR), cephalosporin (J01D) and macrolide (J01F) along with the higher rate of the consumption of broad-spectrum antibiotics (J01 B/N). Negative, inhibitor, the correlation was recorded with a narrow-spectrum penicillin group (J01CE, J01CF). In female lung cancer cases, the comparison has yielded opposite associations: significant inhibitor (negative) correlation was observed with broad-spectrum penicillin (J01CA, J01CR), cephalosporin (J01D) and quinolone (J01M), while a supportive relationship was observed with narrow-spectrum penicillin (J01CE, J01CF).

### 4.3. Melanoma

The statistical analyses resulted in several, positive and negative associations; it did not show any difference between the male and female cases. Melanoma incidence was inversely associated with the total consumption of antibiotics (J01) in both sexes and protective (inhibitor) association was detected between broad-spectrum penicillin combined with a beta-lactamase inhibitor (J01CR), but not with broad-spectrum, beta-lactamase sensitive penicillin (J01CA), which raises the possible protective effect of beta-lactamase inhibitors, most likely clavulanic acid, on the development of melanoma. Similarly, the inhibitor effect was associated with cephalosporin (J01D) and quinolone (J01M) consumption. Strong supportive significance was recorded between melanoma incidence and the consumption of narrow-spectrum penicillin (J01CE, J01CF).

### 4.4. Breast

An only a weak positive correlation was observed with the narrow-spectrum, beta-lactamase-resistant penicillin (J01CF, *p* = 0.059).

### 4.5. Prostate

Positive significance was seen with the narrow-spectrum, beta-lactamase-resistant penicillin (J01CF). A statistically insignificant similar association was observed when J01CE and CF were calculated together. Negative significance was found with cephalosporin (J01D) and quinolone (J01M).

### 4.6. Uterus Corpus

A weak, negative association was observed with the narrow-spectrum, beta-lactamase-resistant penicillin (J01CF).

### 4.7. Kidney

Inverse, negative, significance was detected between total antibiotic consumption (J01) and the incidence of kidney cancer in both sexes. A weak, positive correlation was found between broad-spectrum, beta-lactamase sensitive penicillin (J01CA) and negative with narrow-spectrum, beta-lactamase-resistant penicillin (J01CF) in male, kidney cancer patients. Female patients showed a statistically insignificant, negative association with the consumption of quinolone (J01M) and broad-spectrum antibiotics.

### 4.8. Bladder

A positive, significant, correlation was recorded with the joint consumption of broad-spectrum penicillin (J01CA + J01CR), but none, with the separate groups (J01CA and J01CR separately). A statistically insignificant, positive correlation was found with the consumption of cephalosporin (J01D) and quinolone (J01M). No, any association was observed in female cases.

## 5. Discussion

Gut microbiota is composed of different bacteria species taxonomically classified by genus, family, order and phyla. Gut microbiota consists of not only bacteria, but also viruses, fungi and Archaea. Each individual is provided with a unique gut microbiota profile that plays many specific functions in host nutrient metabolism, maintenance of structural integrity of the gut mucosal barrier, immunomodulation and protection against pathogens, etc. [19,20].

Gut microbiota is shaped in early life as their composition depends on infant transitions (birth gestational date, type of delivery, methods of milk feeding, weaning period) and external factors such as antibiotic use. This personal and healthy core native microbiota remains relatively stable in adulthood, but differs between individuals due to enterotypes, body mass index (BMI) level, exercise frequency, lifestyle and cultural and dietary habits and by gender [19,20].

Dysbiosis or disruption of the normal human microbiota is associated with a wide range of diseases, including inflammatory bowel disease, multiple sclerosis, obesity, autism, depression, cardiovascular disease and allergy, as well as cancer [21,22,23].

The microbiome has been implicated in cancer in a variety of specific ways, including being directly oncogenic, through the promotion of oncogenic mucosal inflammation or systemic metabolic/immune dysregulation and through modulation of anti-cancer immunity or the efficacy of anticancer therapy. Bacterial species are found in tumor tissue itself, normal tissue adjacent to the tumor and at tumor sites such as the gut, genitourinary tract and airway, with overlap between these sites [9]. The highest risk was found in individuals with a long duration of antibiotic exposure or those receiving higher doses. There was a 30% increased incidence of lung, hematological, pancreatic and genitourinary cancers compared to controls due to increased antibiotic exposure [5]. An overall increase of 18% for all malignancies [5]. An extensive meta-analysis showed evidence that antibiotic use slightly increases the risk of hematological (multiple myeloma and lymphoma), gastrointestinal (colorectal, hepatobiliary, pancreatic and gastric cancers), lung and genitourinary cancers (prostate, bladder and kidney). Weak evidence supported the increased risk for breast and other cancers such as gynecological cancers and melanoma. Moderate evidence was found that this risk is associated with specific classes of antibiotics (macrolides, beta-lactams, quinolones, sulfonamides and cephalosporins), but low or insufficient evidence of associations with the other analyzed classes [5]. The extensive use of antibiotics may predict the development of cancer [24]. Even maternal antibiotic exposure showed an association with cancer morbidity in children [25]. Our observations (Table 9.) indicated the possible role of different antibiotics in the development of certain malignancies, probably through the induction of dysbiosis in the gut flora or by modifying the composition of tissue bacteria. Sex differences, observed in our analyses, may be associated with the gender-related difference of the gut flora [26]. Although the microbiome influences carcinogenesis through mechanisms independent of inflammation and immune system, the most recognizable link is between the microbiome and cancer via the immune system, as the resident microbiota plays an essential role in activating, training and modulating the host immune response. In certain cases, mechanisms that are more detailed were observed. The interaction between *F. nucleatum* Fap2 protein and host polysaccharide (Gal-GalNAc) mediates *F. nucleatum* colonization in colorectal cancer. *F*. *nucleatum* mediates tumor-immune evasion via the T-cell immunoreceptor with Ig and ITIM domains (TIGIT). The Fap2 protein secreted by *F. nucleatum* interacts with TIGIT and inhibits natural killer (NK) cell–mediated immunosurveillance of cancer [27].

Our concept is further supported by the fact that the rank order (decreasing) of cancer prevalence in countries included in the study and the rank order (decreasing) of the consumption of different antibiotic classes showing positive correlation with the cancer incidence is very similar. The inverse rank order of antibiotics (increasing), showing negative (inhibitor) correlation related to the development of certain cancer types, strengthens the possibility that the less consumption of “inhibitor” antibiotics may increase the incidence of certain malignancies (Table 3, Table 4, Table 5, Table 6, Table 7 and Table 8).

## 6. Conclusions

Our findings strongly support the observations of the role of antibiotics in the development of various malignancies, probably acting through the modification of microbiome and hence, the dominant antibiotic consumption patterns in different countries are reflected in the cancer prevalence data of the given country. Countries with relatively high consumption of narrow-spectrum penicillin (J01CE, J01CF) and tetracycline (J01A), like certain Scandinavian countries, showed a higher incidence of female colorectal cancer, female lung cancer, melanoma, breast, prostate, uterus corpus. Countries with relatively higher consumption of broad-spectrum penicillin (J01CA, J01CR) and some broad-spectrum antibiotics (J01D, J01F, J01M), like Greece, Hungary, Slovakia, France, etc. showed a higher incidence rate of male lung cancer and male bladder cancer. Certain cancers did not show any significance with any classes of antibiotics, like colorectal cancer of males and bladder cancer in females.

Our study included the eight most common cancers in males and females, but further analyses may uncover other possible associations between carcinoma incidence and antibiotic consumption.

## 7. Weakness of the Study

The positive and negative correlation between cancer incidence and antibiotic consumption data could not be applied to the individual level and no other confounding circumstances could be identified, which may influence the results.

## 8. Strengths of the Study

The positive and negative correlation between the large databases of cancer prevalence and antibiotic consumption strongly supports the role of antibiotics in carcinogenesis as described in the literature. The rank order of cancer incidence in different countries are similar to the rank order of antibiotic consumptions. The higher incidence of different cancers shows higher consumptions patterns of antibiotics with “promoting” effect and lower consumption patterns of antibiotics with “inhibitory” effects on cancer incidence.

## Figures and Tables

**Table 1:** Incidence of the most frequent cancer types as per 100,000 inhabitants/country (male/M/and female/F/) in 30 European countries for 2018.

	Colorectum		Lung		Melanoma		Breast	Prostate	Uterus	Kidney		Bladder	
**Countries**	**M**	**F**	**M**	**F**	**M**	**F**	**F**	**M**	**F**	**M**	**F**	**M**	**F**
Austria	39.7	23.9	48.4	33	20.4	15.9	96.2	90.9	2.7	5.6	2.7	8.1	2
Belgium	65.7	41.6	78.1	39.7	21.6	29.7	154.7	96.7	3.3	5.4	2.5	9.1	2.2
Bulgaria	56.2	30	71.1	15.8	6.2	4.8	79.3	82.2	5.3	6.3	1.6	10.3	2.5
Croatia	68.8	36.9	75.6	25.8	12.6	9.2	93.6	80.8	5.2	9.9	3.1	12.8	3.3
Cyprus	52.3	22.9	61.3	12.4	7	5.4	110.5	114.6	4.9	4.8	1.5	11.5	2.3
Czech Republic	63.8	36.7	57.7	26.5	18.8	16.2	97	128.8	4.3	10.6	4.3	8.6	2.5
Denmark	69.5	54.7	56.4	53.8	30.3	41.7	121.1	111.7	3.8	6.1	2.4	8.3	3.2
Estonia	53.3	39	76.3	21.9	12.7	15	83.1	162.4	4.2	12.1	4.2	8.7	1.8
Finland	43.4	31.5	37.8	21.8	22.9	20.7	122.9	108.4	3.9	6	2.6	4.1	0.9
France	55.3	36.7	74.2	31.8	19.1	16.7	133.3	144.9	4	7	2.7	10.1	1.9
Germany	46.6	32.8	60.9	39.2	26.9	29.9	116.2	94.4	2.7	7.5	3.1	8.1	2.3
Greece	48.6	31.1	99	23.5	10	12.8	94.3	76	4.1	5.9	1.8	12.9	1.5
Hungary	104.2	54.1	111.6	58.7	14	13.2	116	90.4	4.4	7.8	4	10.8	3.1
Iceland	45.8	33.5	41	48.1	11	14.3	116.7	86.9	2	7.3	3.4	5	0.8
Ireland	64.2	39.6	59.4	43.9	19.2	25.2	123.2	189.3	4.9	5.7	2.7	6.3	3.1
Italy	54.3	36.7	52.7	23.3	18.1	13.7	125.4	91	3.1	4.8	2	8.3	1.7
Latvia	64.6	41.1	77.6	14	8.9	7.2	85.1	121.2	7.6	11.9	4	16.9	2
Lithuania	53.5	32.6	77	13.5	12.1	12.1	80.6	97.9	5.6	13	4.1	9.5	1.5
Luxembourg	48.2	37.8	60.9	26.6	24.9	19.2	148.8	116.7	3.1	2.7	2	8.1	1.6
Malta	54.8	34.3	43.7	17.1	10.4	10.9	121	88.3	4.8	6.3	2.3	7.7	2.2
Netherlands	68.1	45.9	52	47.1	37	33.5	143.8	101.2	3.4	6.8	3	8.2	2.4
Norway	71.2	58.8	47	43.3	41.1	40.8	118.7	157.3	3.9	5.2	2.2	6.6	1.7
Poland	61.3	32.8	78.5	35.4	8.2	6.5	79.5	65.6	5.7	8.6	3.4	14.9	2.7
Portugal	80.2	42.1	55.2	13.8	9.7	8.3	94	87.7	2.8	3.8	1.6	9.1	1.9
Romania	53.5	28.1	72.8	18.1	4.5	4.6	70.3	47.2	3.2	4.8	1.9	9.7	1.7
Slovakia	90.3	46	79.7	19.1	13.6	10.5	81.8	78.3	6.1	10.9	4.9	13.4	2.4
Slovenia	87.7	37.6	68.5	29.7	24.7	25.2	93.4	117.2	5	8.9	2.7	8.2	2.2
Spain	67.7	34.4	62.3	19.7	7.4	9.4	101.2	104.2	3.5	5.7	1.8	11.2	1.8
Sweden	44.9	36.4	25.6	26.4	32.9	34.1	122.9	149.8	3.2	4.7	2.6	5.9	2
UK	56.7	39.6	55	45.6	21.2	20.1	127.7	120.9	4.1	5.8	2.6	7.3	3.7

**Table 2:** Average, yearly antibiotic consumption in 30 European countries for 1997–2018 (22 years) derived from the ECDC yearly reports. Total antibiotic consumption for systemic use (J01), expressed as defined daily dose (DDD)/1000 inhabitants/day (DID). Other classes of antibiotics at ATC Level 3 and 4 (for the penicillin group/J01C/) consumed has been featured as the relative share of the total consumption and estimated in percentage (%).

Antibiotic Consumption ECDC 1997–2018	100% J01 (DID)	J01A (%)	J01C (%)	J01CA (%)	J01CR (%)	J01CA+CR (%)	J01CE (%)	J01CF (%)	J01CE+CF (%)	J01D (%)	J01F (%)	J01M (%)	J01 B/N 2018
Austria	12.12	9.21	35.74	6.92	20.7	27.54	8.23	0.07	8.3	13.14	26.71	11.56	6.62
Belgium	21.96	11.33	40.07	17.3	21.03	38.31	0.44	1.17	1.6	11.12	14.63	10.79	122.41
Bulgaria	17.39	13.34	37.45	23.45	9	32.46	5.07	0.1	5.16	14.71	14.28	11.18	49.6
Croatia	18.59	7.9	42.19	13.58	22.7	36.28	5.73	0.16	5.9	17.72	15.06	8.35	11.94
Cyprus	26.95	11.76	35.17	11.34	23.58	34.93	0.34	0.09	0.43	21.53	11.61	16.27	37.96
Czech Republic	15.01	15.12	36.71	6.66	13.03	22.36	12.76	0.43	13.19	8.29	19.89	7.36	no data
Denmark	14.18	9.84	62.59	19.01	2.51	21.34	33.53	7.5	41.03	0.2	14.9	3.37	0.59
Estonia	10.41	20.8	32.88	20.29	9	29.3	2.44	0.06	2.5	9.42	19.39	8.07	15.95
Finland	16.79	23.89	29.75	15.87	4.5	20.37	9.23	0.4	9.62	13.85	10.35	5.31	0.48
France	24.88	13.04	44.13	26.85	15.74	41.6	0.72	1.46	2.17	11.37	16.61	7.87	37.16
Germany	12.9	20.99	27.39	16.65	2.38	19.03	8.5	0.11	8.61	16.82	19.02	9.94	6.53
Greece	30.42	8.26	27.81	13.44	12.79	26.17	1.62	0.01	1.63	24.59	27.75	8.92	624.04
Hungary	14.96	10.68	35.96	10.26	21.1	31.36	4.59	0	4.59	14.28	20.77	12.64	68.27
Iceland	19.47	25.46	48.23	17.47	11.58	29.06	12.99	5.95	18.94	2.57	8.19	4.19	1.53
Ireland	18.25	2.8	44.98	14.84	18.88	33.73	5.05	5.98	11.04	8.49	18.9	5.01	4.45
Italy	22.01	10.65	42.45	16.45	25.53	41.99	0.05	0.07	0.12	12.7	21.78	14.75	226.82
Latvia	10.58	22.15	38.1	26.4	10.9	37.31	0.93	0.02	0.95	4.87	13.1	9.5	19.67
Lithuania	15.89	10.6	48.14	31.75	8.46	40.22	7.63	0.41	8.04	9	12.04	6.74	7.79
Luxembourg	23.01	9.94	34.97	13.42	20.04	33.33	0.42	0.87	1.29	18.62	18.17	10.96	68.22
Malta	18.83	6.53	33.5	3.15	30.26	33.42	0.39	0.27	0.66	23.24	20.58	11.55	80.32
Netherlands	9.34	25.57	32.12	13.72	10.44	24.17	4.06	3.65	7.72	0.6	14.74	9.18	20.25
Norway	15.3	19.38	40.57	12.83	0	12.68	24.16	3.42	27.58	1.02	10.92	3.42	0.16
Poland	18.77	14.46	33.7	20.94	12.03	32.33	2.21	0.11	2.32	12.78	18.23	6.82	25.48
Portugal	18.34	5.74	42.35	11.9	27.67	39.57	0.16	3.21	3.38	12.67	17.48	14.25	50.82
Romania	24.14	4.1	47.17	18.12	23.19	41.32	3.1	2.6	5.71	19.03	11.51	13.06	18.44
Slovakia	21.52	8.56	39.41	10.5	15.14	25.62	13.46	0.08	13.54	16.87	22.06	8.93	11.15
Slovenia	13.19	4.06	55.4	16.6	21.78	23.52	15.66	1.1	16.76	3.83	19.39	9.89	3.12
Spain	17.26	5.79	51.11	20.62	28.44	49.07	0.58	1.28	1.86	11.25	14.7	13.8	56.33
Sweden	13.81	22.01	47.45	7.59	1.3	8.17	28.68	9.53	38.21	2.32	5.5	6.23	0.21
United Kingdom	15.259	25.78	38.397	21.37	4.95	26.32	4.859	7.149	12.008	3.965	17.298	3.622	1.77

ATC codes: J01A—tetracycline; J01C—penicillin; J01CA—broad-spectrum, beta-lactamase sensitive penicillin; J01CR—broad-spectrum penicillin combined with beta-lactamase inhibitors; J01CE—narrow-spectrum, beta-lactamase sensitive penicillin; J01CF—narrow-spectrum, beta-lactamase-resistant penicillin; J01D—cephalosporin; J01F—macrolide; J01M—quinolone; J01 B/N—ratio of broad-spectrum and narrow-spectrum antibiotics.

**Table 3:** Rank order (decreasing) of the incidence of male lung cancer (10 countries) compared to the rank order (decreasing) of antibiotics with suspected “promoter” effect and the increasing (reverse) rank order of the suspected “inhibitor” effect of antibiotics. Of the first ten countries with the highest incidence rate of male lung cancer, nine countries can be found among the highest consumption figures of “promoter” antibiotics (exception: Poland). Seven countries with the lowest consumption figures of “inhibitor” antibiotics, are among the first high incidence rate of male lung cancer (exception: Hungary, Lithuania, Estonia)

	Incidence of Lung Cancer (Male) in Decreasing Rank Order	Rank Order (Decreasing) of Antibiotics with Possible “Promoting” Effect on the Development of Lung Cancer					
Countries	New Cases/100,000 Inhabitants 2018	Countries	J01CA + J01CR (%)	Countries	J01D (%)	Countries	J01F (%)
Hungary	111.6	Spain	49.07	Greece	24.59	Greece	27.75
Greece	99	Italy	41.99	Malta	23.24	Austria	26.71
Slovakia	79.7	France	41.6	Cyprus	21.53	Slovakia	22.06
Poland	78.5	Romania	41.32	Romania	19.03	Italy	21.78
Belgium	78.1	Lithuania	40.22	Luxembourg	18.62	Hungary	20.77
Latvia	77.6	Portugal	39.57	Croatia	17.72	Malta	20.58
Lithuania	77	Belgium	38.31	Slovakia	16.87	Czech Rep	19.89
Estonia	76.3	Latvia	37.31	Germany	16.82	Estonia	19.39
Croatia	75.6	Croatia	36.28	Bulgaria	14.71	Slovenia	19.39
France	74.2	Cyprus	34.93	Hungary	14.28	Germany	19.02
	**Incidence of Lung Cancer (Male) in Decreasing Rank Order**	**Rank Order (Increasing) of Antibiotics with Possible “Inhibitor” Effect on the Development of Lung Cancer**					
**Countries**	**New cases/100,000 inhabitants 2018**	**Countries**	**J01CE + J01CF (%)**			**Countries**	**J01A (%)**
Hungary	111.6	Italy	0.12			Ireland	2.8
*Greece*	99	Cyprus	0.43			Slovenia	4.06
*Slovakia*	79.7	Malta	0.66			Romania	4.1
*Poland*	78.5	Latvia	0.95			Portugal	5.74
*Belgium*	78.1	Luxembourg	1.29			Spain	5.79
*Latvia*	77.6	Belgium	1.6			Malta	6.53
Lithuania	77	Greece	1.63			Croatia	7.9
Estonia	76.3	Spain	1.86			Greece	8.26
*Croatia*	75.6	France	2.17			Slovakia	8.56
*France*	74.2	Poland	2.32			Austria	9.21

J01CA—broad-spectrum, beta-lactamase sensitive penicillin; J01R—broad-spectrum penicillin with beta-lactamase inhibitors; J01D—cephalosporin; J01F—macrolide; J01CE—narrow-spectrum, beta-lactamase-sensitive penicillin; J01CF—narrow-spectrum, beta-lactamase-resistant penicillin; J01A—tetracycline.

**Table 4:** Rank order (decreasing) of the incidence of female lung cancer (10 countries) compared to the rank order (decreasing) of antibiotics with suspected “promoter” effect and the increasing (reverse) rank order of suspected “inhibitor” effect of antibiotics. Out of the first ten countries with the highest incidence rate of female lung cancer, five countries can be found among the highest consumption figures of “promoter” antibiotics (bold, italics). Eight countries with the lowest consumption figures of “inhibitor” antibiotics are among the firs high incidence rate of female lung cancer (bold, italics).

Incidence of Lung Cancer (Female) in Decreasing Rank Order		Rank Order (Decreasing) of Antibiotics with Possible “Promoting” Effect on the Development of Lung Cancer		Incidence of Lung Cancer (Female) in Decreasing Rank Order		Rank Order (Increasing) of Antibiotics with Possible “Inhibiting” Effect on the Development of Lung Cancer					
Countries	New Cases/100,000 Inhabitants 2018	Countries	J01CE+CF	Countries	New Cases/100,000 Inhabitants. 2018	Countries	J01CA + CR (%)	Countries	J01D (%)	Countries	J01M (%)
Hungary	58.7	Denmark	41.03	Hungary	58.7	Sweden	8.17	Denmark	0.2	Denmark	3.37
*Denmark*	53.8	Sweden	38.21	*Denmark*	53.8	Norway	12.68	Norway	0.6	Norway	3.42
*Iceland*	48.1	Norway	27.58	*Iceland*	48.1	Germany	19.03	United Kingdom	1.02	United Kingdom	3.622
Netherlands	47.1	Iceland	18.94	*Netherlands*	47.1	Finland	20.37	Iceland	2.32	Iceland	4.19
*United Kingdom*	45.6	Slovenia	16.76	*United Kingdom*	45.6	Denmark	21.34	Ireland	2.57	Ireland	5.01
*Ireland*	43.9	Slovakia	13.54	*Ireland*	43.9	Czech Republic	22.36	Finland	3.83	Finland	5.31
*Norway*	43.3	Czech Rep	13.19	*Norway*	43.3	Slovenia	23.52	Sweden	3.965	Sweden	6.23
Belgium	39.7	United Kingdom	12.008	Belgium	39.7	Netherlands	24.17	Lithuania	4.87	Lithuania	6.74
Germany	39.2	Ireland	11.04	*Germany*	39.2	Slovakia	25.62	Poland	8.29	Poland	6.82
Poland	35.4	Finland	9.62	*Poland*	35.4	Greece	26.17	Czech Republic	8.49	Czech Rep	7.36

J01CE—narrow-spectrum, beta-lactamase-sensitive penicillin; J01CF—narrow-spectrum, beta-lactamase-resistant penicillin; J01CA—broad-spectrum penicillin; beta-lactamase sensitive penicillin; J01CR—broad-spectrum penicillin with beta-lactamase inhibitors; J01D—cephalosporin; J01M—quinolone.

**Table 5:** Rank order (decreasing) of the incidence of breast cancer (10 countries) compared to the rank order (decreasing) of antibiotics with suspected “promoter” effect. Of the first ten countries with the highest incidence rate of breast cancer, seven countries can be found among the highest consumption figures of “promoter” antibiotics (bold. italics).

	Incidence of Breast Cancer in Decreasing Rank Order	Rank Order (Decreasing) of Antibiotics with Possible “Promoting” Effect on the Development of Breast Cancer			
Countries	New Cases/100,000 Inhabitants. 2018	Countries	J01A (%)	Countries	J01CF (%)
Belgium	154.7	United Kingdom	25.78	Sweden	9.53
Luxembourg	148.8	Netherlands	25.57	Denmark	7.5
*Netherlands*	143.8	Iceland	25.46	United Kingdom	7.149
*France*	133.3	Finland	23.89	Ireland	5.98
*United Kingdom*	127.7	Latvia	22.15	Iceland	5.95
Italy	125.4	Sweden	22.01	Netherlands	3.65
*Ireland*	123.2	Germany	20.99	Norway	3.42
*Finland*	122.9	Estonia	20.8	Portugal	3.21
*Sweden*	122.9	Norway	19.38	Romania	2.6
*Denmark*	121.1	Czech Republic	15.12	France	1.46

J01CF—narrow-spectrum, beta-lactamase-resistant penicillin; J01A—tetracycline.

**Table 6:** Rank order (decreasing) of the incidence of prostate cancer (10 countries) compared to the rank order (decreasing) of antibiotics with suspected “promoter” effect. Of the first ten countries with the highest incidence rate of prostate cancer, six countries can be found among the highest consumption figures of “promoter” antibiotics (bold. italics). Similarly, seven countries with the lowest consumption figures of “inhibitor” antibiotics are among the firs high incidence rates of prostate cancer (bold. italics).

The Incidence of Prostate Cancer in Decreasing Rank Order		Rank Order (Decreasing) of Antibiotics with Possible “Promoting” Effect on the Development of Prostate Cancer		The Incidence of Prostate Cancer Is Decreasing Rank Order		Rank Order (Increasing) of Antibiotics with Possible “Inhibiting” Effect on the Development of Prostate Cancer					
Countries	New Cases/100,000 Inhabitants. 2018	Countries	J01CE + CF (%)	Countries	New Cases/100,000 Inhabitants. 2018	Countries	J01CA + CR (%)	Countries	J01D (%)	Countries	J01M (%)
*Ireland*	189.3	Denmark	41.03	*Ireland*	189.3	Sweden	8.17	Denmark	0.2	Denmark	3.37
Estonia	162.4	Sweden	38.21	Estonia	162.4	Norway	12.68	Netherlands	0.6	Norway	3.42
*Norway*	157.3	Norway	27.58	*Norway*	157.3	Germany	19.03	Norway	1.02	United Kingdom	3.62
*Sweden*	149.8	Iceland	18.94	*Sweden*	149.8	Finland	20.37	Sweden	2.32	Iceland	4.19
France	144.9	Slovenia	16.76	France	144.9	Denmark	21.34	Iceland	2.57	Ireland	5.01
*Czech Republic*	128.8	Slovakia	13.54	*Czech Republic*	128.8	Czech Republic	22.36	Slovenia	3.83	Finland	5.31
Latvia	121.2	Czech Republic	13.19	*Latvia*	121.2	Slovenia	23.52	United Kingdom	3.965	Sweden	6.23
*United Kingdom*	120.9	United Kingdom	12.008	*United Kingdom*	120.9	Netherlands	24.17	Latvia	4.87	Lithuania	6.74
*Slovenia*	117.2	Ireland	11.04	*Slovenia*	117.2	Slovakia	25.62	Czech Republic	8.29	Poland	6.82
Luxembourg	116.7	Finland	9.62	Luxembourg	116.7	Greece	26.17	Ireland	8.49	Czech Republic	7.36

J01CE—narrow-spectrum, beta-lactamase-sensitive penicillin; J01CF—narrow-spectrum, beta-lactamase-resistant penicillin; J01CA—broad-spectrum, beta-lactamase sensitive penicillin; J01CR—broad-spectrum penicillin with beta-lactamase inhibitors; J01D—cephalosporin; J01M—quinolone.

**Table 7:** Rank order of melanoma in males (decreasing) compared to the rank order (decreasing) of antibiotic consumptions of “promoting” and the rank order (increasing) of “inhibitor” effect of antibiotics. Eight countries with the highest consumption of “promoting” antibiotics included in the first ten highest incidences of melanoma (males) countries. Eight of ten countries with the lowest consumption of “inhibitory” antibiotics are included in the first ten countries with the highest incidence of melanoma (males).

Incidence of Melanoma (Male) in Decreasing Rank Order		Rank Order (Decreasing) of Antibiotics with Possible “Promoting” Effect on the Development of Lung Cancer				Incidence of Melanoma (Male) in Decreasing Rank Order		Rank Order (Increasing) of Antibiotics with Possible “Inhibiting” Effect on the Development of Melanoma					
Countries	New Cases/100,000 Inhabitants. 2018	Countries	J01A	Countries	J01CE + CF	Countries	New Cases/ 100,000 Inhabitants2018	Countries	J01CA + CR (%)	Countries	J01D (%)	Countries	J01M (%)
*Norway*	41.1	United Kingdom	25.78	Denmark	41.03	*Norway*	41.1	Sweden	8.17	Denmark	0.2	Denmark	3.37
*Netherlands*	37	Netherlands	25.57	Sweden	38.21	*Netherlands*	37	Norway	12.68	Norway	0.6	Norway	3.42
*Sweden*	32.9	Iceland	25.46	Norway	27.58	*Sweden*	32.9	Germany	19.03	United Kingdom	1.02	United Kingdom	3.622
*Denmark*	30.3	Finland	23.89	Iceland	18.94	*Denmark*	30.3	Finland	20.37	Iceland	2.32	Iceland	4.19
*Germany*	26.9	Latvia	22.15	Slovenia	16.76	*Germany*	26.9	Denmark	21.34	Ireland	2.57	Ireland	5.01
Luxembourg	24.9	Sweden	22.01	Slovakia	13.54	Luxembourg	24.9	Czech Republic	22.36	Finland	3.83	Finland	5.31
Slovenia	24.7	Germany	20.99	Czech Rep	13.19	*Slovenia*	24.7	Slovenia	23.52	Sweden	3.965	Sweden	6.23
*Finland*	22.9	Estonia	20.8	United Kingdom	12.008	*Finland*	22.9	Netherland	24.17	Lithuania	4.87	Lithuania	6.74
Belgium	21.6	Norway	19.38	Ireland	11.04	Belgium	21.6	Slovakia	25.62	Poland	8.29	Poland	6.82
*United Kingdom*	21.2	Czech Republic	15.12	Finland	9.62	*United Kingdom*	21.2	Greece	26.17	Czech Republic	8.49	Czech Rep	7.36

J01A—tetracycline; J01CE—narrow-spectrum, beta-lactamase sensitive penicillin; J01CF—narrow-spectrum, beta-lactamase-resistant penicillin; J01CA—broad-spectrum, beta-lactamase sensitive penicillin; J01CR—broad-spectrum, beta-lactamase inhibitor penicillin; J01D—cephalosporin; J01M—quinolone.

**Table 8:** Rank order of melanoma in females (decreasing) compared to the rank order (decreasing) of antibiotic consumptions of “promoting” effect and the rank order (increasing) with “inhibitor” effect of antibiotics. Eight countries with the highest consumption of “promoting” antibiotics included in the first ten highest incidences of female melanoma countries. Nine of ten countries with the lowest consumption of “inhibitory” antibiotics are included in the first ten countries with the highest incidence of melanoma (females).

Incidence of Melanoma (Female) in Decreasing Rank Order		Rank Order (Decreasing) of Antibiotics with Possible “Promoting” Effect on the Development of Lung Cancer			Incidence of Melanoma (Female) in Decreasing Rank Order					Rank Order (Increasing) of Antibiotics with Possible “Inhibiting” Effect on the Development Melanoma			
Countries	New Cases/100,000 Inhabitants 2018	Countries	J01A	Countries	J01CE + CF	*Countries*	New Cases/100,000 Inhabitants 2018	Countries	J01CA + CR (%)	Countries	J01D (%)	Countries	J01M (%)
***Denmark***	41.7	United Kingdom	25.78	Denmark	41.03	***Denmark***	41.7	Sweden	8.17	Denmark	0.2	Denmark	3.37
***Norway***	40.8	Netherlands	25.57	Sweden	38.21	***Norway***	40.8	Norway	12.68	Norway	0.6	Norway	3.42
***Sweden***	34.1	Iceland	25.46	Norway	27.58	***Sweden***	34.1	Germany	19.03	United Kingdom	1.02	United Kingdom	3.622
***Netherlands***	33.5	Finland	23.89	Iceland	18.94	***Netherlands***	33.5	Finland	20.37	Iceland	2.32	Iceland	4.19
***Germany***	29.9	Latvia	22.15	Slovenia	16.76	***Germany***	29.9	Denmark	21.34	Ireland	2.57	Ireland	5.01
Belgium	29.7	Sweden	22.01	Slovakia	13.54	Belgium	29.7	Czech R.	22.36	Finland	3.83	Finland	5.31
Ireland	25.2	Germany	20.99	Czech Rep	13.19	***Ireland***	25.2	Slovenia	23.52	Sweden	3.965	Sweden	6.23
***Slovenia***	25.2	Estonia	20.8	United Kingdom	12.008	***Slovenia***	25.2	Netherlands	24.17	Lithuania	4.87	Lithuania	6.74
***Finland***	20.7	Norway	19.38	Ireland	11.04	***Finland***	20.7	Slovakia	25.62	Poland	8.29	Poland	6.82
***United Kingdom***	20.1	Czech Republic	15.12	Finland	9.62	***United Kingdom***	20.1	Greece	26.17	Czech Republic	8.49	Czech R.	7.36

J01A—tetracycline; J01CE—narrow-spectrum, beta-lactamase sensitive penicillin; J01CF—narrow-spectrum, beta-lactamase-resistant penicillin; J01CA—broad-spectrum, beta-lactamase sensitive penicillin; J01CR—broad-spectrum, beta-lactamase inhibitor penicillin; J01D—cephalosporin; J01M—quinolone.

**Table 9:** Summary of correlation and significance (Pearson’s) between antibiotic consumption and major types of cancers (male, female). Positive significance: marked with yellow filling color, negative significance: marked with green, statistically insignificant (positive, negative, *p* = 0.05–0.09) marked with orange filling color.

**Antibiotics** **Pearson’s**		**Colorectum**		**Lung**		**Melanoma**		**Breast**	**Prostate**	**Uterus Corpus**	**Kidney**		**Bladder**	
		M	F	M	F	M	F	F	M	F	M	F	M	F
J01	*r*	−0.160	−0.328	0.261	−0.279	−0.414	−0.382	0.055	−0.307	−0.077	−0.388	−0.432	0.199	−0.218
	*p*	0.399	0.076	0.164	0.135	0.023	0.037	0.773	0.099	0.686	0.034	0.017	0.291	0.248
J01A	r	−0.327	0.111	−0.350	0.270	0.381	0.309	0.259	0.226	−0.114	0.154	0.283	−0.250	−0.146
	*p*	0.077	0.560	0.058	0.148	0.038	0.096	0.167	0.230	0.549	0.416	0.130	0.182	0.443
J01C	*r*	0.266	0.246	−0.150	0.130	0.076	0.219	−0.059	0.125	−0.049	−0.015	−0.096	−0.136	0.090
	*p*	0.156	0.190	0.429	0.493	0.690	0.246	0.755	0.509	0.799	0.936	0.615	0.474	0.635
J01CA	*r*	−0.100	−0.052	0.304	−0.087	−0.218	−0.143	−0.206	0.027	0.294	0.351	0.098	0.262	−0.092
	*p*	0.600	0.786	0.102	0.646	0.248	0.451	0.275	0.886	0.115	0.057	0.608	0.163	0.630
J01CR	*r*	0.244	−0.264	0.203	−0.324	−0.525	−0.547	−0.051	−0.320	−0.036	−0.216	−0.279	0.231	−0.001
	*p*	0.194	0.159	0.281	0.081	0.003	0.002	0.789	0.084	0.851	0.251	0.135	0.220	0.994
J01CA+CR	*r*	0.068	−0.287	0.389	−0.373	−0.695	−0.664	−0.163	−0.302	0.133	0.016	−0.174	0.418	−0.063
	*p*	0.721	0.124	0.034	0.042	<0.001	<0.001	0.389	0.105	0.483	0.933	0.358	0.021	0.741
J01CE	*r*	0.094	0.427	−0.397	0.368	0.605	0.673	0.040	0.271	−0.110	0.029	0.141	−0.374	0.090
	*p*	0.621	0.019	0.030	0.045	<0.001	<0.001	0.832	0.147	0.563	0.881	0.459	0.042	0.637
J01CF	*r*	−0.074	0.311	−0.525	0.463	0.451	0.561	0.349	0.379	−0.344	−0.349	−0.167	−0.506	0.197
	*p*	0.698	0.095	0.003	0.010	0.012	0.001	0.059	0.039	0.062	0.059	0.377	0.004	0.296
J01CE+CF	*r*	0.059	0.434	−0.464	0.424	0.617	0.701	0.123	0.322	−0.180	−0.066	0.073	−0.440	0.125
	*p*	0.757	0.017	0.010	0.020	<0.001	<0.001	0.517	0.083	0.342	0.727	0.701	0.015	0.510
J01D	*r*	−0.162	−0.513	0.369	−0.491	−0.577	−0.611	−0.209	−0.488	0.074	−0.155	−0.242	0.333	−0.098
	*p*	0.392	0.004	0.045	0.006	0.001	<.0.001	0.269	0.006	0.699	0.413	0.198	0.072	0.607
J01F	*r*	0.170	−0.044	0.426	0.032	−0.152	−0.192	−0.122	−0.168	0.052	0.074	0.103	0.262	0.230
	*p*	0.368	0.819	0.019	0.866	0.422	0.310	0.520	0.374	0.783	0.699	0.587	0.163	0.221
J01M	*r*	0.119	−0.358	0.248	−0.479	−0.468	−0.537	−0.135	−0.422	−0.073	−0.249	−0.351	0.335	−0.112
	*p*	0.530	0.052	0.187	0.007	0.009	0.002	0.477	0.020	0.700	0.184	0.057	0.070	0.555
J01 B/N	*r*	−0.131	−0.142	0.387	−0.136	−0.207	−0.157	0.016	−0.267	−0.076	−0.208	−0.318	0.250	−0.215
	*p*	0.491	0.455	0.035	0.473	0.272	0.407	0.934	0.153	0.688	0.270	0.087	0.183	0.253
